# Investigation of cardiovascular characteristics in a patient with hereditary hemorrhagic telangiectasia, a case report

**DOI:** 10.14814/phy2.70987

**Published:** 2026-06-21

**Authors:** Søren Søndergaard, S. Madsen

**Affiliations:** ^1^ Centre of Planned Surgery, Regional Hospital of Silkeborg & Institute of Clinical Medicine University of Aarhus Aarhus Denmark; ^2^ Department of Cardiology Aarhus University Hospital Aarhus Denmark

**Keywords:** cardiovascular functions, case report, heart rate variability, Mb. Osler‐Weber‐Rendu disease, total blood volume

## Abstract

Hereditary Hemorrhagic Telangiectasia (HHT) may present with symptoms arising from multiple organ systems. In this case report, the focus is on cardiovascular manifestations, including total blood volume, cardiac function, and the potential role of the sympathetic nervous system (SNS) in the clinical picture. The commonly held supposition that the cardiac hyperdynamic state in HHT is associated with increased SNS activity is examined by combining spectral analysis of heart rate variability with carbon monoxide–based measurement of total blood volume and transthoracic echocardiography. The observations are consistent with Guytonian venous return physiology and suggest a circulation characterized by low venous return resistance.

## 1 INTRODUCTION

Hereditary Hemorrhagic Telangiectasia (HHT), also known as Osler‐Weber‐Rendu disease, is a genetically determined vascular disorder. It is characterized by spontaneous and relapsing epistaxis, multiple telangiectasias in typical locations such as the lips, oral cavity, fingers, and nose, and visceral telangiectasias or arteriovenous malformations in the lungs, liver, brain, and spinal cord.

A substantial proportion of patients develops hepatic involvement with liver or portal shunting, leading to a hyperdynamic circulatory state with high cardiac output that may progress to high‐output cardiac failure. It is often assumed that this state is associated with a predominance of sympathetic tone in the autonomic balance, although, to our knowledge, this has not been substantiated by frequency‐domain heart rate variability (HRV) analyses in HHT. It has also been proposed that activation of the renin–angiotensin–aldosterone system (RAAS) contributes to increased blood volume via angiogenesis, but this has not been clearly documented in the available literature (Farhan et al., 2022; Kritharis et al., 2018). This report describes a 63‐year‐old woman with HHT who underwent excision of a cerebellar hemangioblastoma and was subsequently admitted to the neurorehabilitation unit at the Regional Hospital in Silkeborg. HHT was considered a concurrent condition rather than the primary reason for admission. The investigations summarized below therefore represent the most extensive cardiovascular assessment feasible within the constraints of this setting. Patient characteristics are presented with the patient's permission.

### 1.1 Patient information

**FIGURE 1:**
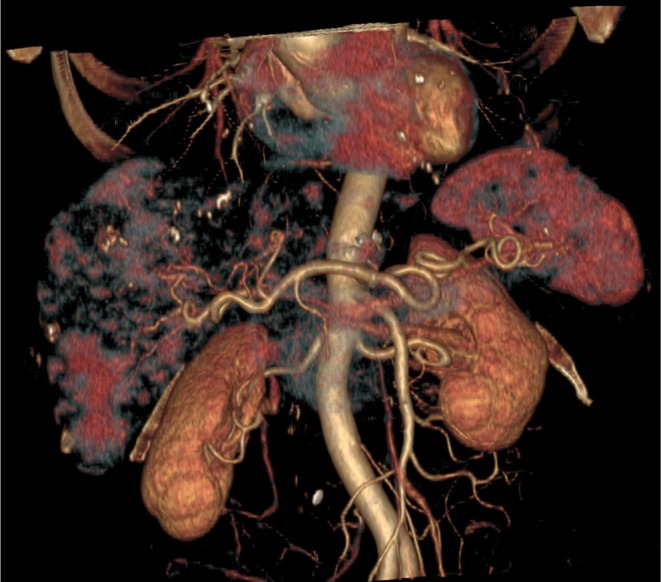
3D reconstruction of a CT scan with intravenous contrast of the upper abdomen. Large confluent vascular masses are seen in all lobes of the liver (The picture is brought with the patient's permission and an acknowledgement to Vejle Hospital Department of Radiology, which has made the reconstruction available to us).

The principal HHT‐related symptoms were intermittent epistaxis and orthostatic intolerance with orthostatic hypotension. Postoperative sequelae included dysphagia, tongue paresis, diplopia, and partial Parinaud syndrome with impaired upward gaze. She had no relevant medical history unrelated to HHT and had not undergone interventional or surgical treatment for HHT apart from recurrent nasal cauterisation at an otorhinolaryngology department. This case report focuses on visceral lesions that manifest as arteriovenous fistulas and their cardiovascular impact; see Figure 1. The completed CARE checklist has been provided as a Appendix S1.

### 1.2 Cardiac output (Qs)

Transthoracic echocardiography demonstrated a moderately dilated left atrium and a hyperdynamic left ventricle with an ejection fraction above 65%. Stroke volume was estimated at approximately 90 mL from LVOT measurements (LVOT diameter 2 cm, LVOT VTI 29 cm), at a heart rate of 95 beats/min, yielding a cardiac output (Qs) of 8.5–9 L/min. There was no echocardiographic evidence of pulmonary hypertension based on estimated systolic pulmonary artery pressure. For the patient's age group, a Qs of 3.9 ± 1.06 L/min is considered normal; the measured Qs therefore corresponded to approximately double the age‐adjusted mean Qs (Slotwiner et al., 1998), see Table 1.

**TABLE 1:** Summary of paraclinical assessments with patient results and reference values, mean and SD.

Paraclinical assessment	Parameter	Patient result	Reference value
Echocardiography	Cardiac output	8.5–9 L/min	3.9 ± 1.06 L/min
Heart rate variability	Low and High Frequency power, n.u.	LF power 48% ± 7.7% HF power 52% ± 7.6%	LF power 55%–65% ± 15% HF power 35%–45% ± 15%.
CO measurement	TBV PV Hb mass	TBV 5.5 L (+17%) PV 3.6 L (+28%) Hb mass, 651 g (+13%)	TBV: 3.7–5.7 L PV: 2.1–3.5 L Hb mass 458–698 g

### 1.3 HRV

Cardiac autonomic modulation was assessed using a smartphone‐based seismocardiography application (Kubios, Kuopio, Finland) (Lipponen et al., 2026). The algorithm is based on spectral analysis of the intervals between apex beats, recorded by the phone's accelerometer at a sampling frequency of 200 Hz (depending on hardware). Output is provided for selected linear, non‐linear, and spectral indices, including RR interval, SDNN, Poincaré SD1 and SD2, LF, HF, and the LF/HF ratio. The frequency spectrum is traditionally decomposed into low‐frequency (LF) and high‐frequency (HF) bands. LF is often interpreted as reflecting mixed sympathetic and parasympathetic modulation, whereas HF is considered to mirror predominantly parasympathetic (vagal) modulation, although this interpretation is debated (Malik, 1996; Notarius et al., 1999; Sammito et al., 2024). Five measurements were obtained over a two‐hour period. LF power (normalized units, n.u.) was 48 ± 7.7%, and HF power (n.u.) was 52 ± 7.6%. While there is no universal consensus on normal values, recent normative data for healthy women aged 60–74 years report LF power (n.u.) of approximately 55%–65% ± 15% and HF power (n.u.) of 35%–45% ± 15% (Brinth et al., 2024). In this patient, these findings did not show a clear spectral shift towards sympathetic predominance. Remaining variables were: SDNN 7.59 ± 3.2 ms, Poincaré SD1 5.89 ± 1.82 ms, and Poincaré SD2 9.0 ± 2.73 ms. See Table 1.

### 1.4 TBV

The patient's total blood volume (TBV) was determined using the Detalo Clinical™ device, based on carbon monoxide (CO) rebreathing (Breenfeldt Andersen et al., 2023; Detalo, 2026). The patient was connected to a closed‐circuit system, and a bolus of 0.7 mL CO/kg body weight was administered during tidal ventilation. CO was rebreathed for 4 min, with simultaneous CO_2_ absorption and supplemental O_2_ to maintain pressure and volume in the circuit, after which the patient was disconnected and breathed room air. The remaining CO in the measuring device was not measured. In other studies, residual CO in the system has typically been reported in the range of 50–300 ppm, corresponding to an overestimation of hemoglobin mass by up to 20 g, which we note as a potential minor source of bias. Carboxyhemoglobin was measured before CO administration and 2 min after disconnecting from the system. Because CO has an affinity for hemoglobin that is 200–250 times greater than that of oxygen and the inhaled O_2_:CO ratio was approximately 15–20, it is reasonable to assume that essentially all inhaled CO was bound to hemoglobin, with negligible free CO in the blood. TBV was calculated as 5.5 L [reference range: 3.7–5.7 L; +17%], and plasma volume was 3.6 L [reference range: 2.1–3.5 L; +28%], based on total hemoglobin mass, hemoglobin concentration, and hematocrit (Breenfeldt Andersen et al., 2023; Siebenmann et al., 2017). See Table 1.

### 1.5 Treatment

Orthostatic hypotension was managed with gradual mobilization from supine to sitting and upright positions, with ambulation supported in a walking frame. An oral α‐adrenergic vasoconstrictor was trialed but did not result in a clinically meaningful improvement in blood pressure. The vasoconstrictor may primarily have affected the native vascular bed rather than the neoangiogenic vascular network, thereby redistributing blood between the two vascular compartments without improving effective orthostatic tolerance. Postoperative (hemorrhagic) and secondary anemia were treated with iron supplementation and transfusion of 500 mL erythrocyte concentrate.

## 2 DISCUSSION

The cardiovascular profile of this patient is consistent with a high‐output circulatory state. In HHT, widespread arteriovenous anastomoses reduce systemic vascular resistance and the resistance to venous return, thereby increasing venous return and cardiac output. This interpretation has therapeutic implications, as it suggests that hypervolemia and a hyperdynamic circulation in patients with Mb. Osler may be driven primarily by a low‐resistance vascular pathology (Singh, 2023). August Krogh famously stated that “the heart cannot do more than send out what it gets” (Krogh, 1912). This aligns with the Guytonian framework, in which a normally functioning left ventricle is not the principal determinant of cardiac output; rather, cardiac output is largely governed by the venous return gradient and systemic venous capacitance. Patterson and Starling demonstrated in heart–lung preparations that, at constant resistance and heart rate, cardiac output is primarily determined by venous pressure—that is, the venous return gradient—an intrinsic property of the myocardium that persists even in denervated hearts (Guyton, 1968; Patterson & Starling, 1914). Magder epitomized this Guytonian understanding of the cardiovascular determinants thus: “The real role of the heart in regulating cardiac output is to lower right atrial pressure and allow better drainage from the compliant veins and venules. This also means that it is not left heart function and left atrial pressure which are the major determinants of cardiac output, but rather right heart function and right atrial pressure” (Magder, 1998). In parallel with the familiar expression Qs = (MAP – CVP)/SVR, where MAP is mean arterial pressure, CVP is central venous pressure, and SVR is systemic vascular resistance, venous return (VR) can be described as VR = (MSFP – CVP)/RVR, where MSFP is mean systemic filling pressure and RVR is resistance to venous return. In HHT, RVR is likely reduced by arteriovenous fistulas that lie largely beyond central nervous control, and VR is further augmented by elevated blood volume, which increases MSFP. CVP thereby becomes a co‐determinant of blood flow through its role in the venous return gradient (MSFP–CVP) and is, in turn, influenced by cardiac function and venous return (Gelman, 2008). Taken together, these considerations support the interpretation that the high‐output state in HHT is primarily driven by a low‐resistance vascular pathology.

### 2.1 Therapeutic consequences

The coexistence of a native vascular circuit and a disease‐related parallel circuit may help explain why vasopressor therapy did not improve orthostatic hypotension in this case. Similarly, abrupt restoration of vascular resistance by liver transplantation or extensive embolisation of fistulas, without simultaneous management of expanded blood volume, may pose a hemodynamic risk by suddenly re‐establishing a major resistance element in the venous compartment.

## AUTHOR CONTRIBUTIONS

**Søren Søndergaard:** Conceptualization; data curation; formal analysis; investigation; methodology; project administration; software; validation. **S. Madsen:** Formal analysis; methodology; validation.

## FUNDING INFORMATION

The manuscript received no funding.

## CONFLICT OF INTEREST STATEMENT

The authors have no COIs to disclose.

## ETHICS STATEMENT

Not applicable, all measurements were made within a diagnostic and therapeutic rationale.

## PATIENT CONSENT

The patient provided verbal consent, witnessed by the next of kin and physicians in the neurorehabilitative ward and, at a later stage, in writing.

## PERMISSION TO REPRODUCE MATERIAL FROM OTHER SOURCES

Permission has been obtained from the Radiological Dept., Vejle Hospital on the condition of reference.

## Supporting information


**Appendix S1.** CARE checklist.

## Data Availability

Data are available upon reasonable request.
