# Supplementary material for: Investigation of cardiovascular characteristics in a patient with hereditary hemorrhagic telangiectasia, a case report
Source: Physiol Rep. 2026 Jun 21;14(12):e70987. doi: 10.14814/phy2.70987 (PMC13284087; doi:10.14814/phy2.70987)
Supplement: Supplementary file 1 — Appendix S1. CARE checklist. [file PHY2-14-e70987-s001.pdf]

CARE checklist item	Manuscript line number(s) Morbus-Osler
1. Title – diagnosis/intervention + "case report"	1–4
2. Keywords – 2–5 key words incl. "case report"	22–23
3a. Abstract – what is unique/added to literature	27–33
3b. Abstract – main symptoms/important clinical findings	27–33, 51–57
3c. Abstract – main diagnoses, interventions, outcomes	35–47, 108–113, 115–118
3d. Abstract – main take-away lessons	115–118
4. Introduction – why case is unique (may include references)	35–46, 47–48
5a. Patient information – de-identified patient-specific information	47–49
5b. Patient information – primary concerns and symptoms	51–53
5c. Patient information – medical, family, psycho-social, genetic information	35–36, 53–56
5d. Patient information – relevant past interventions with outcomes	47–48, 55–56
6. Clinical findings – significant PE and clinical findings	52–53
7. Timeline – historical and current episode of care	47–48, 51–57, 108–113
8a. Diagnostic assessment – diagnostic testing (PE, labs, imaging, etc.)	59–6[illegible], 71–7[illegible], 8[illegible]–91, 9[illegible]–10[illegible], Table 1 (around 115–118)
8b. Diagnostic assessment – diagnostic challenges	Not reported
8c. Diagnostic assessment – diagnosis and other diagnoses considered	35–41, 115–117
8d. Diagnostic assessment – prognosis (e.g., staging)	142–145
9a. Therapeutic intervention – types (pharmacologic, surgical, preventive, self-care)	47–48, 108–113
9b. Therapeutic intervention – administration (dose, strength, duration)	96–103, 112
9c. Therapeutic intervention – changes and rationale	108–113
10a. Follow-up and outcomes – clinician/patient-assessed outcomes	59–66, 83–91, 95–106, 115–118, Table 1
10b. Follow-up and outcomes – important follow-up tests	Table 1 (paraclinical summary, ~115–118)
10c. Follow-up and outcomes – adherence and tolerability	108–110
10d. Follow-up and outcomes – adverse/unanticipated events	52–53
11a. Discussion – strengths and limitations of case report	115–118, 119–145
11b. Discussion – relevant literature with references	39–46, 119–145, References 164–195
11c. Discussion – rationale for conclusions, possible causes	115–118, 129–141
11d. Discussion – primary take-away lessons (one-paragraph conclusion)	115–118
12. Patient perspective	Not reported
13. Informed consent – whether patient gave consent (YES)	YES
